# Factors influencing disclosure among women experiencing intimate partner violence during pregnancy in Moshi Municipality, Tanzania

**DOI:** 10.1186/s12889-016-3345-x

**Published:** 2016-08-04

**Authors:** Victor Katiti, Geofrey Nimrod Sigalla, Jane Rogathi, Rachel Manongi, Declare Mushi

**Affiliations:** 1 Kilimanjaro Christian Medical University College, P.O Box 2240, Moshi, Tanzania; 2 Evangelical Lutheran Church in Tanzania, P.O Box 3033, Arusha, Tanzania; 3 Kilimanjaro Christian Medical Center, P.O Box 2240, Moshi, Tanzania

**Keywords:** Partner violence during pregnancy, IPV disclosure, Risk and protective factors

## Abstract

**Background:**

Intimate Partner Violence (IPV) has serious negative health effects to millions of women around the globe. While disclosing IPV could open doors for support and eventually prevent partner abuse, the factors associated with IPV disclosure during pregnancy are not well known. The aim of this study was to examine factors influencing IPV disclosure to any person of interest or organization supporting women during pregnancy in Moshi Municipality, Tanzania.

**Methods:**

Data were from a prospective cohort study of 1123 pregnant women followed-up by the project aiming to assess the impact of violence in the reproductive health conducted in Moshi Municipality, Tanzania from March 2014 to May 2015. Inclusion criteria to the current analysis were all 339 pregnant women who reported to have experienced physical, sexual and/or emotional violence during the index pregnancy. Data analysis used SPSS Version 20. Odds ratio with 95 % Confidence Interval (CI) for factors associated with IPV disclosure was estimated using multivariate logistic regression models while controlling for age, education and parity. A p-value of less than 0.05 was considered for a statistically significant difference.

**Results:**

IPV disclosure was found to be 23.3 % (*n* = 79). Disclosure of IPV was less likely among unemployed (OR = 0.5, 95 % CI 0.30–0.90) and women whose index pregnancy was unplanned (OR = 0.53, 95 % CI 0.29–0.98). Women who regularly participated in women’s or community groups, religious groups or political associations at least once a month had 2 times higher odds of IPV disclosure compared to those who did not attend regularly (OR = 2.12, 95 % CI 1.13–3.95). Most of the abused women during pregnancy who disclosed their experience of IPV (69 %) disclosed to a member of the family of birth followed by friends (14 %) and a member of family of the partner (11 %).

**Conclusions:**

Most of the women who experienced IPV during pregnancy kept suffering in silence while less than a quarter of all the abused (23.3 %) disclosed their experience to someone. Identification of the women experiencing IPV during pregnancy should be done as a starting point for supporting victim of IPV. Women empowerment in economical and reproductive health will reduce their vulnerability and facilitate disclosure of IPV for support. Key individuals who informally support victims of IPV should be targeted in interventions.

**Electronic supplementary material:**

The online version of this article (doi:10.1186/s12889-016-3345-x) contains supplementary material, which is available to authorized users.

## Background

Intimate partner violence (IPV) is a serious public health problem globally. IPV is the most common type of violence against women perpetuated by men and estimated to be around 30 % globally [1]. IPV prevalence is as high as 39 % in East Africa [1], 44 % in Tanzania [2] and 21 to 31 % in Moshi [3]. Studies documents IPV related consequences such as increased risk of injury, stress, HIV infection, death, separation and reduced contraceptive use [1, 2, 4]. IPV related risks during pregnancy include spontaneous abortion, premature labor, preterm birth and delivery of low birth weight infants [5–7].

IPV disclosure may result into positive impacts to the victims if the process of disclosure is taken care well. The reported positive impacts of IPV disclosure include stop of further violence, safety of pregnant women and their pregnancy and assisting in the creation of new interventions towards violence [8, 9]. Also disclosure can be one of the means of survival from violence.

Despite the high prevalence of IPV to the general population and during pregnancy, disclosure of IPV experience has remained low globally. Studies that tried to explore IPV disclosure have estimated to happen among 4 to 8 % globally [10], 8.8 % in Tanzania and 1.0 % in Kilimanjaro [2]. On the other hand, most of the published studies on IPV focus on general population of women than they do during pregnancy. Pregnancy creates opportunity to recognize and support women with IPV; however, women themselves need to be willing to freely share their experience. Understanding factors influencing disclosure of IPV during pregnancy may have strong implication in planning for future interventions. The objective of this study was to determine factors influencing disclosure of intimate partner violence experienced by women during pregnancy in Moshi Municipality, Tanzania.

## Methods

This study was carried out within a larger study titled “The Impact of Violence on Reproductive Health in Tanzania and Vietnam (PAVE)”. The study was a prospective cohort and recruited a total of 1123 pregnant women when they were attending antenatal care in two health care facilities in Moshi District –Tanzania from March 2014 to May 2015. Women who fulfilled the inclusion criteria were enrolled in the study, interviewed at baseline and followed up at 34 weeks gestation for the second interview. Only women with singleton pregnancy, at gestational age below 24 weeks based on ultrasound scan and willing to be followed up for the entire period of the study were enrolled in the study.

After consenting participation, first interview was conducted using questionnaire to capture baseline socio-demographic and reproductive health characteristics of the participants (Additional file 1). Follow up interview was done at week 34 of pregnancy to assess exposure to IPV in the index pregnancy (Additional file 2). All interviews were conducted in a private room at the clinic and each lasted for 45 to 60 min. Only the research assistant and the participant were allowed to be present. Information was collected through face to face interviews in *Swahili* language.

Our current study extracted information of all 339 pregnant women who reported to have experienced emotional, physical and/or sexual violence during pregnancy (Additional file 3). We did a cross sectional analysis of data collected at Majengo and Pasua antenatal clinics of Moshi Municipality between April and June 2015.

### Measures

The tool on assessing exposure to IPV was adopted form the WHO Multi-Country Study on Women Health and Domestic Violence against Women Kiswahili version and was used in Tanzania before [11]. Women were considered to have experienced any form of IPV during pregnancy if they reported any of the acts for physical (have been hit, slapped, kicked, physically hurt, or threatened with any weapon), emotional (insulted, humiliated, intimidated or threatened) and/or sexual violence (physically forced to have sexual intercourse, sexual intercourse without freely given consent or forced to do a humiliating or degrading sexual act). Women were further asked additional question as to whether (yes/no) and from whom they sought help to try end violence.

Independent variables were socio-demographic characteristics (age, level of education, occupation and marital status), reproductive history (parity, pregnancy intention as women’s personal subjective feeling regarding their current pregnancy whether the timing of conception was planned or not) and health risk behavior of alcohol consumption during pregnancy. Detailed social characteristics were inquired and included place of growth (this community or another community), living close to the family of birth and/or the family of the partner (yes/no), frequency of talking to a member of family of birth and/or of the partner (at least once in a week, once in a month or in a year or never) and whether they counted support from family of birth and/or of the partner in case of problems (yes/no). Socialization was also inquired as to whether they participated in women’s or community groups, religious groups or political associations at least once a month (yes/no).

Data were analyzed using Statistical Package for social Sciences (SPSS, version 20.0, 2011). Descriptive statistics including frequency and proportion for baseline characteristics were done. On bivariate analysis, socio-demographic and reproductive health characteristics among those who did or did not disclose were compared using odds ratio (ORs) with 95 % confidence interval (CIs). Multivariate logistic regression analysis was done by including all factors which were significant in the bivariate logistic regression analysis to determine the most significant factors for IPV disclosure and also control for confounders. A factor was considered a confounder when a change to crude OR after adjustment was 10 % or more. P-value of less than 0.05 was considered for a statistical significant difference.

## Results

The mean age of participants was 26 years (Standard deviation SD = 5.8 years). Their age ranged from 18 to 44 years. The overall characteristics of respondents are displayed in Table 1. Majority of participants 259 (76.4 %) were between 20 and 34 years of age, had primary school education 209 (61.7 %) and were employed 231(68.1 %). More than three quarter of the participants 283(83.5 %) did not use any alcohol during pregnancy and nearly the same proportion 248 (73.2 %) reported the timing of the current pregnancy at the time of conception was planned.

**Table 1:** Demographic and reproductive health characteristics of pregnant women who reported to have experienced any form of IPV (*n* = 339)

Characteristic	Number of women, n	Percentage, %
Age (years)		
18–19	39	11.5
20–34	259	76.4
35 and above	41	12.1
Education level		
Primary and below	209	61.7
Secondary and above	130	38.3
Marital status		
Married & living together	288	85.0
Have partner but living apart	51	15.0
Occupation		
Employed^a^	231	68.1
Unemployed	108	31.9
Parity		
None	115	33.9
1–2	168	49.6
3–7	56	16.5
Any alcohol use during pregnancy		
Ever use	56	16.5
Never use	283	83.5
Planned pregnancy		
Yes	248	73.2
No	91	26.8

^a^employed = those with formal employment and self employed

As shown in Table 2, most of the participants 230 (67.8 %) had migrated to Moshi town. Slightly more than half of the participants (56.0 %) lived close to the family of the partner and 227 (67.0 %) lived close to the family of birth. Out of 339, 191 (56.4 %) women were in frequent communication with a member of family of birth. Equal proportions of the remaining half 74(21.8 %) either communicated with any member of the family of birth at least once a month or never at all. On the other hand, nearly half 164 (48.4 %) of the participants were able to talk to any member in the family of in-laws at least weekly while nearly three in ten 93 (27.4 %) did it rarely or never.

**Table 2:** Socio - demographic characteristics of pregnant women who reported to have experienced any form of IPV (*n* = 339)

Characteristic	Number of women, n	Percentage, %
Place of growth		
This community	109	32.2
Another community	230	67.8
Living close to family of birth		
Yes	227	67.0
No	112	33.0
Living close to family of the partner		
Yes	190	56.0
No	149	44.0
Talk to a member of family of birth		
At least once in a week	191	56.4
At least once in a month	74	21.8
At least once in a year or never	74	21.8
Talk to a member of family of the partner		
At least once in a week	164	48.4
At least once in a month	82	24.2
At least once in a year or never	93	27.4
Count on a member of family of birth for support		
Yes	257	75.8
No	82	23.2
Count on a member of family of the partner for support		
Yes	219	64.6
No	120	35.4
Attending group, organization or association		
Yes	57	16.8
No	282	83.2

Over one third (35.4 %) of women would never depend on support from a member of family of the partner as compared to nearly a quarter 82 (23.2 %) who did the same to a member of the family of birth. Most of the participants in the study 282 (83.2 %) were not attending any support group or organizations.

### IPV disclosure

Out of 339 pregnant women who experienced any form of IPV, 79 (23.3 %) reported to have disclosed IPV to someone. As Table 3 shows, higher proportion of women who disclosed for IPV were aged 35 years and above, living with partner, employed and with 3 or more previous deliveries. Higher proportions of IPV disclosure were also noted among those who were not prepared for the index pregnancy and those who regularly attended women’s or community groups, religious groups or political associations. Fairly equal proportions of disclosure are observed with regard to either living proximity to or frequency of communication with members of family of birth and of the partner.

**Table 3:** Socio-demographic and reproductive health factors associated with IPV disclosure (*n* = 339)

Characteristic	Total number, n	Disclosed IPV n (%)	Crude OR (95 % CI)	*P* -value	Adjusted OR (95 % CI)^ab^	*P* -value
Age (years)						
18–19	39	6 (15.3)	1.0			
20–34	259	61 (23.6)	1.69 (0.68–4.24)	*0.259*	*-*	*-*
35 and above	41	12 (29.3)	2.28 (0.76–6.84)	*0.143*		
Education level						
Primary and below	209	46(22)	1.0			
Secondary and above	130	33(25.3)	1.21(0.72–2.01)	*0.475*	*-*	
Marital status						
Married & living together	288	68(23.6)	1.0			
Have partner but living apart	51	11(21.6)	0.89 (0.43–1.83)	*0.751*	*-*	
Occupation						
Employed	231	62(26.8)	1.0		1.0	
Unemployed	108	17(15.7)	0.51(0.28–0.92)	*0.026*	0.53(0.30–0.90)	*0.019*
Parity						
None	115	21(18.3)	1.0			
1–2	168	41(24.4)	1.45 (0.80–2.61)	*0.221*	*-*	
3–7	56	17(30.3)	1.95 (0.93–4.09)	*0.077*		
Any alcohol use during pregnancy						
Ever use	56	16(28.5)	1.40(0.73–2.66)	*0.309*	*-*	
Never use	283	63(22.2)	1.0			
Planned pregnancy						
Yes	248	50(20.1)	1.0		1.0	
No	91	29(31.9)	*0.54 (0.32–0.93)*	*0.025*	0.53(0.29–0.98)	*0.042*
Place of growth						
This community	109	25(22.9)	0.97(0.57–1.67)	*0.912*	*-*	
Another community	230	54(23.5)	1.0			
Living close to family of birth						
Yes	227	52 (22.9)	1.0			
No	112	27(24.1)	1.07(0.63*–*1.82)	*0.806*	*-*	
Living close to family of the partner						
Yes	190	46(24.2)	1.0			
No	149	33(22.1)	0.86 (0.52–1.44)	*0.575*	*-*	
Talk to a member of family of birth						
At least once in a week	191	45(23.5)	1.0			
At least once in a month	74	18(24.3)	1.04 (0.56–1.95)	*0.896*	*-*	
At least once in a year or never	74	16(21.6)	0.90 (0.47–1.71)	*0.737*		
Talk to a member of family of the partner						
At least once in a week	164	41(25)	1.0			
At least once in a month	82	21(25.6)	1.03 (0.56–1.90)	*0.917*	*-*	
At least once in a year or never	93	17(18.2)	0.67 (0.36–1.27)	*0.217*		
Count on the family of birth for support						
Yes	257	62(24.1)	1.22(0.66–2.23)	*0.527*	*-*	
No	82	17(20.7)	1.0			
Count on the family of the partner for support						
Yes	257	48(28)	0.81(0.48–1.35)	*0.415*	*-*	
No	82	31(34.9)	1.0			
Attending group, organization or association						
Yes	57	21(36.8)	2.25 (1.22–4.15)	*0.009*	2.12(1.13–3.95)	*0.019*
No	282	58 (20.5)	1.0		1.0	

^a^Include all factors which were significant in the results of crude analysis, *p* < 0.05

^b^Additional adjustment for age, education and parity

### Factors associated with IPV disclosure

Table 3 also presents how socio-demographic and reproductive health factors were associated with IPV disclosure in the univariate logistic regression analysis. Lower odds of IPV disclosure was observed among women who were unemployed compared those who were employed (OR = 0.51, 95 % CI 0.28–0.92). Participants who reported that their pregnancy was unplanned had 46 % lower odds of IPV disclosure compared to their counterpart, who did report appropriate timing (OR = 0.54, 95 % CI 0.32–0.93). On the other hand, participants who attended group, organization or associations had more than 2 times higher odds of IPV disclosure compared to those who did not attend (OR = 2.25, 95 % CI 1.22–4.15).

Results of adjusted analysis in Table 3 show that participants who were unemployed had 47 % lower odds of IPV disclosure compared to those who were employed (OR = 0.53, 95 % CI 0.30–0.90). Again, women who reported that their current pregnancy was unplanned had 47 % lower odds of IPV disclosure compared to those who felt it okay to have current pregnancy (OR = 0.53, 95 % CI 0.29–0.98). However, women who regularly participated in women’s or community groups, religious groups or political associations at least once a month had 2 times higher odds of IPV disclosure compared to those who did not attend (OR = 2.12, 95 % CI 1.13–3.95).

### Disclosure pattern

**Fig. 1:**
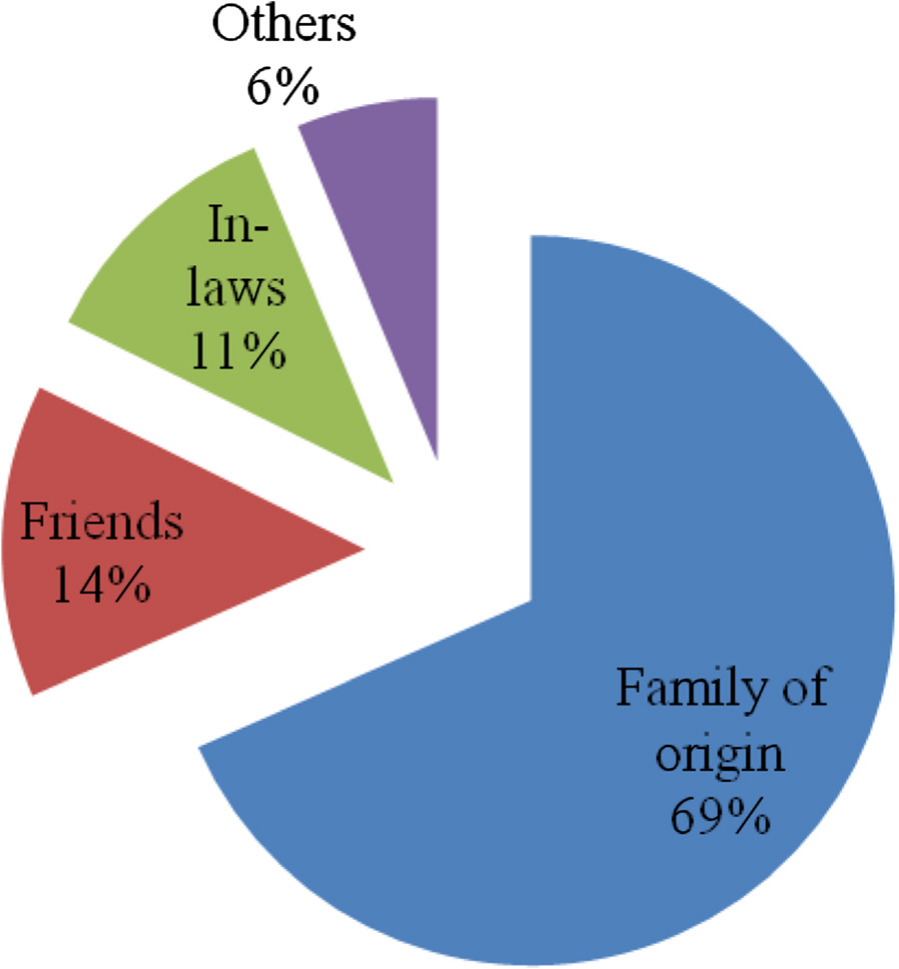
Pie chart showing the pattern of IPV disclosure to a most significant person (*n* = 79)

Disclosure pattern is as displayed in Fig. 1. Most victims of IPV who disclosed, did it to a member of family of origin namely; her own parents, uncle, aunt, brother or sister. Friends were the next likely group to receive information with regard to IPV abuse followed by a member in family of the partner. The rest did disclose to neighbors, police, health worker, religious leaders and women’s, community or religious groups.

## Discussion

This study aimed at examining factors influencing disclosure of IPV experienced by women during pregnancy in Moshi Municipality, Tanzania. To the best of our knowledge, this is the first study in low income countries to determine factors influencing disclosure of IPV abuse during pregnancy in a longitudinal study and at a primary health care setting.

The key findings of this study indicate that, less than a quarter (23 %) of all women who were exposed to physical, sexual and/or emotional abuse during pregnancy disclosed their experience and the rest kept suffering in silence. IPV disclosure was less likely among women who were unemployed and with unplanned pregnancy. Participation in women’s or community groups, religious groups or political associations facilitated IPV disclosure. Abused women during pregnancy preferred to disclose IPV to members of family of origin followed by friends and to the family of in-laws.

This study found that IPV disclosure is very low. Tanzania Demographic Health Survey (TDHS) 2010 [2] assessed lifetime IPV disclosure among ever-partnered women and showed that the prevalence of disclosure was relatively high. About half of women who experienced violence sought help from someone to stop violence, 35 % did not tell anyone and therefore did not ask for help and 10 % did tell someone but never asked for support. The main reason for discrepancies in the rates of disclosure is the fact that TDHS assessed lifetime disclosure of IPV while the current study focused during pregnancy period. Understanding the prevalence of IPV disclosure during pregnancy is important as it will alert policy makers and researchers on the seriousness of the problem [4, 10]. Information on the prevalence of IPV disclosure do assist health educationists, human right activist and other stakeholders in designing targeted interventions to promote IPV disclosure and to stop violence against women [9]. Disclosure of IPV is an essential step for ensuring that victims of violence can seek safe refuge, access supportive services and obtain legal protection [2, 12, 13].

Low level of IPV disclosure could be due to African kingship system, cultural and religious background where family issues are not expected to be exposed outside the marriage/relationships. Women are expected to be submissive to their husbands and disclosing information related to violence outside marriage/relationship is seen as immoral [14]. On the other hand pregnant women may be considering IPV as their own personal problem and as such, cannot be disclosed to other people. Some women also believe that violence will end within a short period of time but unfortunately, the problem persists. Men become loving again after bouts of violence – and this cyclothymic expression of love by husbands few days after violence - creates pseudo hope among women that violence won’t happen again [15]. Hence, they fail to disclose at the right time.

This study revealed that women who were unemployed were less likely to disclose their experience of IPV compared to women who were employed. Pregnant woman who depend on their partner for economic support fail to disclose because the partner may, as a consequence, refuse to support them. In many instances, unemployment is determined by education level and in such cases, education remains as a pro factor to employment. Although education was not significantly associated with IPV disclosure in our study, its link to employment and IPV disclosure cannot be overlooked. Studies have shown that educated women feels more safe, confident and protected when compared to those who are less educated [16]. In addition, educated women are also likely to have formal employment or have capacity to engage in income generating activities and therefore economically independent [16]. Previous study by Bazargan et al. among Malawian women [17] revealed that, women with a higher level of education have higher access to resources and therefore less tolerant of an abusive relationship. Evidence supports that, women with no education are more likely to disclose to the family of origin compared to educated women who disclose even to institutions [18, 19]. Also, unplanned pregnancy remain to be caused by lack of women empowerment in deciding for their own reproductive health [20].

According to previous studies, other factors which influence IPV disclosure are nature/type of the IPV, severity of IPV, having children, personal factors and normalizing violence experiences as an expression of love [15, 21, 22].

This study further revealed that participants who attended women’s organizations were more likely to disclose compare to those who did not attend. The reasons here could be because those who attend organizations are more likely to be exposed to others who might have experienced IPV. They may be encouraged by others to share their life challenges and therefore contribute to the likelihood of IPV disclosure. On the other hand, most of women associations are geared towards economic empowerment and therefore decreasing women vulnerability from economic dependency to their partners [23, 24].

Victims of abuse preferred to disclose their IPV status to a member of the family of origin compared to a member of the family of partner. The majority of women who experienced IPV in this study relied on the informal networks as their first point of contact rather than formal services. Similar pattern of IPV disclosure was reported previously [2]. It could also be a result of discouragement from the community that a husband cannot be taken to institutions and that; issues in the family should not be taken outside the family.

Nearly three quarter of the participants had disclosed IPV to the family of origin. Majority of them preferred their parents, followed by their brothers or sister and uncle or aunt. These findings are similar to the study done in Nigeria where larger proportions of participants (68 %) were willing to disclose their IPV status to the family [18]. The plausible reason as to why IPV victims prefer to disclose to the family of origin could be because of the very strong family ties and the feeling of being more secure; contrary to when they do to other people outside the family. In most African culture, misunderstandings within married couples are supposed to be solved by the family of the partner first and family of origin would come later - especially so when divorce or separation is the course of action. Therefore, disclosure to family of origin may delay action required to settle the conflict. This is one of the important information in designing interventions. Planners should not target the victims or perpetuators only, but the entire family.

Family of the in-laws could play a very significant role to end IPV because of the feeling that it is easy for the parents to face their son. But the situation was different from this study as only 11 % disclosed to the family of in-laws. In African settings, a woman is seen by the in-laws as someone respected. This may hinder victims from facing the in-law to disclose IPV [25].

Less than one in ten participants had disclosed to police, counselor, NGO/women’s organization or local leaders while in Nigeria, 26 % of women exposed to IPV disclosed to formal institutions [14]. Formal institutions such as police, courts, health care facilities, religious and NGOs are expected to be crucial in preventing violence and in supporting women who experience violence and IPV in particular.^.^ One reason for less likelihood of disclosing to institutions would be that they are more formal and women are not sure of what formal action/measures will be taken to stop further violence once they disclose IPV. Primary focus of abused women is bringing back peace in the relationship and the least they would want is family breakdown. This may be the case if men decide to react when appropriate actions are taken to them as perpetrators [22]. Therefore, pregnant women may be afraid of disclosing to such institutions unless IPV becomes more severe. Further, pregnant women may not be aware of the services provided by such institutions. From this study, it is evident that informal institutions play a major role in addressing family conflict although its effectiveness needs to be assessed. Therefore further research should focus on the effectiveness of informal support to women who experience IPV.

Health-care providers are in a unique position to create a safe and confidential environment for facilitating disclosure of violence, while offering appropriate support and referral to other resources and services [1]. One documented key reason explaining why victims of IPV are not likely to disclose to health workers is the victim’s feeling that providers lack adequate time to provide help [26, 27]. In addition, there are no clear formalized procedures in the health care setting for victims of IPV to report acts of violence [27]. Lack of trust to health care providers and lack of time in discussing IPV with healthcare providers are other health system factors reported by pregnant women that limits disclosure. However, it has been argued that with optimal conditions for disclosure, women are more comfortable to disclose IPV to health care providers as they will do with family or in neighborhood [28–30]. Their feeling of being unknown make women consider the health care setting a better place to talk confidentially about IPV. Limiting factor for effective support to women would be the capacity and experience of health care providers in addressing IPV in the clinical setting [6].

### Limitations of the study

The study relied on secondary data which made it difficult to clarify missing information with participants. Violence being among the sensitive topics, interviewed participants may have with-held information or provided socially desirable answers to avoid being deviant to their culture or religious beliefs.

## Conclusions

The disclosure of IPV is complex. The findings presented in this study have shown that very low proportions of women who experienced IPV during pregnancy disclosed their experience. Factors for IPV disclosure includes occupation, planned pregnancy and attending group organization or association. We have also observed that majority of women prefer to disclose to their family of origin. Since disclosure is important for setting up interventions to support victims of IPV, findings of this study need to be used to provide important background for interventions that aim at encouraging IPV disclosure and support of victims of IPV. Patterns of disclosure show that members of family of origin and of the partner are preferred by women who share their IPV experience. Therefore, parents and other family member need to be equipped with knowledge on IPV and proper ways to assist IPV victims after disclosure. Family based violence counseling strategies are imperative. Because discloser to the formal institution is very low, these institutions should be equipped to play their role in address IPV in collaboration with informal networks. For pregnant women to be supported well and in a comprehensive way, identifying women who need support should be done and antenatal care could be the best place to start. Further research is needed to explore how informal institutions are effective in addressing the problem of IPV and in helping victims of IPV.

## Abbreviations

CI, confidence interval; DANIDA, Danish international development agency; HIV, human immunodeficiency virus; IPV, intimate partner violence; NGO, non-governmental organization; OR, odds ratio; SD, standard deviation; SPSS, statistical package for social sciences; TDHS, Tanzania demographic health survey; WHO, World Health Organisation

## Additional files

Additional file 1: Enrollment interview questionnaire. (DOC 210 kb) Additional file 2: Follow-up interview questionnaire. (DOC 186 kb) Additional file 3: Database. (XLS 62 kb)
