# Supplementary material for: Factors influencing disclosure among women experiencing intimate partner violence during pregnancy in Moshi Municipality, Tanzania
Source: BMC Public Health. 2016 Aug 4;16:715. doi: 10.1186/s12889-016-3345-x (PMC4973082; doi:10.1186/s12889-016-3345-x)
Supplement: Additional file 1: — Enrollment interview questionnaire. (DOC 210 kb) [file 12889_2016_3345_MOESM1_ESM.doc]

| ID No........................................  PAVE PROJECT  QUESTIONNAIRE 1:  Inclusion Interview  ‘  *Version 6 – Tanzania- ENGLISH*  JANUARY 31 2014  DATE OF INTERVIEW: day [ ][ ] month [ ][ ] year [ ][ ][ ][ ]   | 000. RECORD THE TIME | Hour [ ][ ] (24 h)  Minutes [ ][ ] | | --- | --- | | 001. Name of interviewer | 002. Name of Clinic/Hospital | |
| --- | --- | --- | --- | --- |

| SECTION 1 THE RESPONDENT | | | | | | | | | | | |
| --- | --- | --- | --- | --- | --- | --- | --- | --- | --- | --- | --- |
| QUESTIONS & FILTERS | | | | | CODING CATEGORIES | | | | | | SKIP  TO |
| If you don’t mind, I would like to start by asking you a little about yourself. | | | | | | | | | | |  |
| 101 | | What year were you born? | | | YEAR [ ][ ][ ][ ]  DON’T KNOW YEAR 9998  REFUSED/NO ANSWER 9999 | | | | | |  |
| 102 | | Where did you grow up?  PROBE: Before age 12 where did you live longest? | | | THIS COMMUNITY/NEIGHBOURHOOD................1  ANOTHER RURAL AREA/VILLAGE........................2  ANOTHER TOWN/CITY......................................... 3  ANOTHER COUNTRY........................................... 4  ANOTHER NEIGHBOURHOOD IN SAME TOWN..................................................................... 5  DON’T KNOW/DON’T REMEMBER....................... 8  REFUSED/NO ANSWER........................................ 9 | | | | | |  |
| 103 | | Do any of your family of birth live close enough by that you can easily see/visit them? | | | YES......................................................................... 1  NO........................................................................... 2  LIVING WITH FAMILY OF BIRTH.......................... 3  DON’T KNOW/DON’T REMEMBER ........................8  REFUSED/NO ANSWER.........................................9 | | | | | |  |
| 104 | | Do any of your family in law live close enough by that you can easily see/visit them? | | | YES........................................................................ 1  NO.......................................................................... 2  LIVING WITH IN LAW FAMILY.............................. 3  DON’T KNOW/DON’T REMEMBER .................... . 8  REFUSED/NO ANSWER..................................... . 9 | | | | | |  |
| 105 | | How often do you see or talk to a member of your family of birth? Would you say at least once a week, once a month, once a year, or never? | | | AT LEAST ONCE A WEEK............................ 1  AT LEAST ONCE A MONTH...................... 2  AT LEAST ONCE A YEAR ...................... 3  NEVER (HARDLY EVER) ...................... 4  DON’T KNOW/DON’T REMEMBER............. 8  REFUSED/NO ANSWER ...................... 9 | | | | | |  |
| 106 | | How often do you see or talk to a member of your family in law? Would you say at least once a week, once a month, once a year, or never? | | | AT LEAST ONCE A WEEK......... ......... 1  AT LEAST ONCE A MONTH ......... ......... 2  AT LEAST ONCE A YEAR ......... ......... 3  NEVER (HARDLY EVER) ......... ..... 4  DON’T KNOW/DON’T REMEMBER......... 8  REFUSED/NO ANSWER ......... .... 9 | | | | | |  |
| 107 | | When you need help or have a problem, can you usually count on members of your family of birth for support? | | | YES ......... ......... ......... ......... ......... .........1  NO ......... ......... ......... ......... ......... .........2  DON’T KNOW/DON’T REMEMBER......... 8  REFUSED/NO ANSWER ......... ......... ......... 9 | | | | | |  |
| 108 | | When you need help or have a problem, can you usually count on members of your family in law for support? | | | YES......... ......... ......... ......... ......... ......... 1  NO......... ......... ......... ......... ......... ......... 2  DON’T KNOW/DON’T REMEMBER......... 8  REFUSED/NO ANSWER ......... ......... .........9 | | | | | |  |
| 109 | | What is the highest level of education that you achieved? MARK HIGHEST LEVEL. | | | NEVER ATTENDED SCHOOL ......... .........1  PRIMARY ......... ......... ......... ......... ......... 2  SECONDARY ......... ......... ......... ......... .....3  ABOVE SECONDARY......... ......... ......... ..4  NUMBER OF YEARS SCHOOLING [ ][ ]  DON’T KNOW/DON’T REMEMBER....... 98  REFUSED/NO ANSWER ......... ......... 99 | | | | | |  |
| 110 | | Apart from your housework, what is your occupation? | | | GOVERNMENT EMPLOYEE ......... ......... ......... 1  PRIVATE COMPANY/ORGANISATION EMPLOYEE ........................................................ 2  FARMER ......... ................. ......... ......... ......... ... 3  BUSINESS ......... ................. ......... ......... ......... 4  STUDENT ......... ................ ......... ......... ......... 5  UNEMPLOYED ......... ............... ......... ......... .....6  OTHER (SPECIFY)....................................... | | | | | |  |
| 111 | Do you regularly attend a group, organization or association?    IF YES:  What kind of group, organization or association?  IF NO, PROMPT:  Organizations like women’s or community groups, religious groups or political associations.  MARK ALL MENTIONED  PROBE IF NECESSARY TO IDENTIFY TYPE OF GROUP  ADD COUNTRY-SPECIFIC CODES | | NO A | | | ⇒ IF NO GO TO 112 | | | | | |
|  |  |  |  | | | 111b. How often do you attend? (ASK ONLY FOR EACH MARKED IN 111B - X) | | | | | |
|  |  |  |  |  |  | At least once a week | At least once a month | At least once a year | Never (hardly ever) | | |
|  |  |  | CIVIC/POLITICAL/ UNION B | | | 1 | 2 | 3 | 4 | | |
|  |  |  | SOCIAL WORK/CHARITABLE C | | | 1 | 2 | 3 | 4 | | |
|  |  |  | SPORTS/ARTS/CRAFTS D | | | 1 | 2 | 3 | 4 | | |
|  |  |  | ECONOMIC/SAVINGS CLUB E | | | 1 | 2 | 3 | 4 | | |
|  |  |  | WOMEN’S ORGANIZATION F | | | 1 | 2 | 3 | 4 | | |
|  |  |  | RELIGIOUS ORGANIZATION G | | | 1 | 2 | 3 | 4 | | |
|  |  |  | OTHER:________________ X | | | 1 | 2 | 3 | 4 | | |
| 112 | I have a question about your relationship. Are you married and live together? Married but living apart? Living together but not married? You and your partner have a sexual relationship and live apart? | | | CURRENTLY MARRIED AND LIVING TOGETHER........1 MARRIED BUT LIVING APART............... ......... ......... 2  LIVING WITH A MAN, NOT MARRIED........... ......... ......3  CURRENTLY HAVING A REGULAR PARTNER (SEXUAL RELATIONSHIP), LIVING APART ......... ........4  REFUSED/NO ANSWER ......... ......... ......... ......... ... 99 | | | | | | ⇒113  ⇒112b  ⇒112b  ⇒112b  ⇒112b | |
| 113a | How many times in your life have you been married and/or lived together with a man?  (INCLUDE CURRENT PARTNER IF LIVING TOGETHER) | | | NUMBER OF TIMES MARRIED/ LIVED TOGETHER......... ......... ......... [ ][ ]  DON’T KNOW/DON’T REMEMBER ......... ......... .........98  REFUSED/NO ANSWER ......... ......... ......... ......... 99 | | | | | |  | |
| 113b | Including yourself, in total, how many wives or partners does your husband live with now as if married? | | | TOTAL NUMBER OF WIVES AND LIVE-IN PARTNERS . . . . . . [ ][ ]  DON'T KNOW . . . . . . . . . . . . . . . . . . ......... ......... ......98 | | | | | |  | |
| 114 | What is your religion? | | | NO RELIGION ......... ......... ......... ......... ......... 0  CATHOLIC ......... ......... ......... ......... ......... .........1  LUTHERAN ......... ......... ......... ......... ......... .........2  PENTECOSTAL ......... ......... ......... ......... ......... .........3  SEVENTH DAY ADVENTIST ......... ......... ......... ......... 4  ANGLICAN ......... ......... ......... ......... ......... ......... ...... 5  MUSLIM ......... ......... ......... ......... ......... ......... ......... 6  TRADITIONAL BELIEFS ......... ......... ......... ......... ..... 7  OTHER (specify) ………………………………………. 8  DON’T KNOW/DON’T REMEMBER......... ......... 98  REFUSED/NO ANSWER ......... ......... ......... ..............99 | | | | | |  | |
| 115 | What tribe do you belong to? | | | CHAGA - ROMBO ......... ......... ......... ......... ......... .. 1  CHAGA - MARANGU......... ......... ......... ......... ........ 2  CHAGA - OLD MOSHI......... ......... ......... ......... ..... 3  CHAGA - URU ......... ......... ......... ......... ......... .........4  CHAGA - KIBOSHO ......... ......... ......... ......... ......... 5  CHAGA - NARUMU ......... ......... ......... ......... ......... 6  CHAGA - MACHAME......... ......... ......... ......... ......... 7  CHAGA - KAHE ......... ......... ......... ......... ......... ..... 8  PARE ......... ......... ......... ......... ......... ......... ......... 9  MASAI ......... ......... ......... ......... ......... ......... ............ 10  HAYA ......... ......... ......... ......... ......... ......... ............ 11  MBULU ......... ......... ......... ......... ......... ......... ............12  SAMBAA ......... ......... ......... ......... ......... ......... ......... 13  Other specify.......................................... ......... ......... 14 | | | | | |  | |

| **SECTION 2 HEALTH** | | | | | | | | | | | | | |
| --- | --- | --- | --- | --- | --- | --- | --- | --- | --- | --- | --- | --- | --- |
| I would now like to ask a few questions about your health and use of health services. | | | | | | | | | | | | | |
| 201 | In general, would you describe your overall physical health as excellent, good, fair, poor or very poor? | EXCELLENT ......... ......... ......... ......... ......... 1  GOOD......... ......... ......... ......... ......... ......... 2  FAIR ......... ......... ......... ......... ......... ......... 3  POOR......... ......... ......... ......... ......... ......... 4  VERY POOR ......... ......... ......... ......... ......... 5  DON’T KNOW/DON’T REMEMBER ......... ......... 8  REFUSED/NO ANSWER ......... ......... ......... 9 | | | | | | | | | | |  |
| 202 | In general, would you describe your overall mental health as excellent, good, fair, poor or very poor? | EXCELLENT ......... ......... ......... ......... ......... 1  GOOD ......... ......... ......... ......... ......... ......... 2  FAIR ......... ......... ......... ......... ......... ......... 3  POOR ......... ......... ......... ......... ......... ......... 4  VERY POOR ......... ......... ......... ......... ......... 5  DON’T KNOW/DON’T REMEMBER ......... ......... ...... 8  REFUSED/NO ANSWER ......... ......... ......... ........9 | | | | | | | | | | |  |
| 203 | Have you ever been diagnosed with the following medical conditions? | Yes  1 | | No  2 | | | Dont know  8 | | Refused/No answer 9 | | | |  |
|  | 1. DIABETES | 1 | | 2 | | | 8 | | 9 | | | |  |
|  | 1. HYPERTENSION | 1 | | 2 | | | 8 | | 9 | | | |  |
|  | 1. EPILEPSY | 1 | | 2 | | | 8 | | 9 | | | |  |
|  | 1. DEPRESSION ( Please probe) | 1 | | 2 | | | 8 | | 9 | | | |  |
|  | 1. HIV/AIDS | 1 | | 2 | | | 8 | | 9 | | | |  |
|  | 1. ANY OTHER DISEASE(specify)........................ | 1 | | 2 | | | 8 | | 9 | | | |  |
| 204 | In the past 4 weeks, have you taken medication (including traditional medication):  To help you calm down or sleep?  To relieve pain?  To help you not feel sad or depressed?  FOR EACH, IF YES PROBE:  How often? Once or twice, a few times or many times? | FOR SLEEP  FOR PAIN  FOR SADNESS | | | NO  1  1  1 | ONCE OR TWICE  2  2  2 | | A FEW TIMES(3-7 days)  3  3  3 | | | MANY TIMES (>7 days)  4  4  4 |  | |
| 205 | The next questions are related to other common problems that may have bothered you in the past 4 weeks. If you had the problem in the past 4 weeks, answer yes. If you have not had the problem in the past 4 weeks, answer no. | |  | | | | | YES  1 | | NO  2 | | If No to all ⇒207  If Yes to any ⇒206 | |
|  | Do you often have headaches? | | HEADACHES | | | | | 1 | | 2 | |  |  |
|  | Is your appetite poor? | | APPETITE | | | | | 1 | | 2 | |  |  |
|  | Do you sleep badly? | | SLEEP BADLY | | | | | 1 | | 2 | |  |  |
|  | Are you easily frightened? | | FRIGHTENED | | | | | 1 | | 2 | |  |  |
|  | Do your hands shake? | | HANDS SHAKE | | | | | 1 | | 2 | |  |  |
|  | Do you feel nervous, tense or worried? | | NERVOUS | | | | | 1 | | 2 | |  |  |
|  | Is your digestion poor? | | DIGESTION | | | | | 1 | | 2 | |  |  |
|  | Do you have trouble thinking clearly? | | THINKING | | | | | 1 | | 2 | |  |  |
|  | Do you feel unhappy? | | UNHAPPY | | | | | 1 | | 2 | |  |  |
|  | Do you cry more than usual? | | CRY MORE | | | | | 1 | | 2 | |  |  |
|  | Do you find it difficult to enjoy your daily activities? | | NOT ENJOY | | | | | 1 | | 2 | |  |  |
|  | Do you find it difficult to make decisions? | | DECISIONS | | | | | 1 | | 2 | |  |  |
|  | Is your daily work suffering? | | WORK SUFFERS | | | | | 1 | | 2 | |  |  |
|  | Are you unable to play a useful part in life? | | USEFUL PART | | | | | 1 | | 2 | |  |  |
|  | Have you lost interest in things that you used to enjoy? | | LOST INTEREST | | | | | 1 | | 2 | |  |  |
|  | Do you feel that you are a worthless person? | | WORTHLESS | | | | | 1 | | 2 | |  |  |
|  | Has the thought of ending your life been on your mind? | | ENDING LIFE | | | | | 1 | | 2 | |  |  |
|  | Do you feel tired all the time? | | FEEL TIRED | | | | | 1 | | 2 | |  |  |
|  | Do you have uncomfortable feelings in your stomach? | | STOMACH | | | | | 1 | | 2 | |  |  |
|  | Are you easily tired? | | EASILY TIRED | | | | | 1 | | 2 | |  |  |
|  | TOTAL SCORE | |  | | | | |  | |  | |  | |
| 206 | Just now we talked about problems that may have bothered you in the past 4 weeks. I would like to ask you how you reacted? | | DID NOTHING ......... .... ........................... 1........  SOUGHT MEDICAL HELP (INCLUSIVE TRADITIONAL PRACTITIONER) ......... ..... 2  DISCLOSED TO A FRIEND ......... .............. 3  THOUGHT OF ENDING YOUR LIFE ..... .. 4 | | | | | | | | | ⇒207 | |
| 207 | In your life, have you ever thought about ending your life? | | YES ......... ......... ......... ...........1  NO ......... ......... ......... ......... 2..........................  DON’T KNOW/DON’T REMEMBER 8  REFUSED/NO ANSWER ......... 9 | | | | | | | | | ⇒209 | |
| 208 | Have you ever tried to take your life? | | YES......... ......... ......... ......... ....1  NO......... ......... ......... .................2  DON’T KNOW/DON’T REMEMBER 8  REFUSED/NO ANSWER............9 | | | | | | | | |  | |
| 209 | I would also like to ask you a few questions about smoking and alcohol. Before this pregnancy, how often did you smoke?  Daily?  Occasionally?  Not at all? | | DAILY ......... ......... ......... .........1  OCCASIONALLY ......... ...............2  NOT AT ALL ......... ......... ....3  DON’T KNOW/DON’T REMEMBER 8  REFUSED/NO ANSWER............9 | | | | | | | | |  | |
| 210 | During this pregnancy, how often did you smoke?  Daily?  Occasionally?  Not at all?  Never | | DAILY ......... ......... ......... .........1  OCCASIONALLY......... ................2  NOT AT ALL ......... ......... ....3  NEVER......... ......... ......... ......... 4  DON’T KNOW/DON’T REMEMBER 8  REFUSED/NO ANSWER......... 9 | | | | | | | | |  | |
| 211 | Before this pregnancy, How often did you drink alcohol? Would you say:  Every day or nearly every day  Once or twice a week  1 – 3 times a month  Occasionally, less than once a month  Never | | EVERY DAY OR NEARLY EVERY DAY 1  ONCE OR TWICE A WEEK......... ......... 2  1 – 3 TIMES IN A MONTH ......... ......... ......... 3  LESS THAN ONCE A MONTH......... ......... 4  NEVER ......... ......... ......... ......... ......... .........5  DON’T KNOW/DON’T REMEMBER......... 8  REFUSED/NO ANSWER ......... ......... .........9 | | | | | | | | |  | |
| 212 | During this pregnancy, how often did you drink alcohol?  Would you say:  Every day or nearly every day  Once or twice a week  1 – 3 times a month  Occasionally, less than once a month  Never | | EVERY DAY OR NEARLY EVERY DAY 1  ONCE OR TWICE A WEEK......... ......... 2  1 – 3 TIMES IN A MONTH ......... ......... ......... 3  LESS THAN ONCE A MONTH......... ......... 4  NEVER ......... ......... ......... ......... ......... .........5  DON’T KNOW/DON’T REMEMBER......... 8  REFUSED/NO ANSWER ......... ......... .........9 | | | | | | | | | If Never ⇒SECTION 3 | |
| 213 | In the past 4 weeks, how many alcoholic drinks did you usually have a day? | | USUAL NUMBER OF DRINKS [ ][ ]  NO ALCOHOLIC DRINKS IN PAST 4 WEEKS 00 | | | | | | | | |  | |

| **SECTION 3 PREGNANCIES AND BIRTHS** | | | | |
| --- | --- | --- | --- | --- |
| Now I would like to ask about all the times you have been pregnant during your life. | | | | |
| 301 | How old were you when you were pregnant for the first time? | AGE ....................................YEARS  DONT REMEMBER/DONT KNOW 98  REFUSED 99 | |  |
| 302 | Including your current pregnancy, how many times have you been pregnant? Include pregnancies that did not end up in a live birth.  PROBE: How many pregnancies were with twins, triplets? | a) TOTAL NO. OF PREGNANCIES. [ ][ ]  b) PREGNANCIES WITH TWINS [ ]  c) PREGNANCIES WITH TRIPLETS [ ] | | If this is the first pregnancy ⇒310 |
| 303 | Have you ever had a pregnancy that ended in an abortion, a miscarriage, or a stillbirth?  PROBE: How many times did you miscarry, how many times did you have a stillbirth, and how many times did you abort? | a) MISCARRIAGES [ ][ ]  b) STILLBIRTHS [ ][ ]  c) ABORTIONS [ ][ ]  IF NONE ENTER ‘00’ | |  |
| 304 | Have you ever given birth? How many children have you given birth to that were alive when they were born? (INCLUDE BIRTHS WHERE THE BABY DIDN’T LIVE FOR LONG) | NUMBER OF CHILDREN BORN [ ][ ]    NONE 00 | |  |
| 305 | Of the times you have given birth how many have been preterm below 37 weeks?  IF YES, PROBE HOW MANY TIMES | a) TOTAL NO. OF PRETERM BIRTH [ ][ ]  b) NO................................................................00  c) DON’T KNOW/DON’T REMEMBER 99 | | If no ⇒307 |
| 306 | If yes how many weeks into your pregnancy were you? | _____________ weeks | |  |
| 307 | Have you ever given birth to a child that weighed less than 2.5 Kg?  IF YES, PROBE HOW MANY TIMES | a) TOTAL NO. OF LOW BIRTH WEIGHT DELIVERIES [ ][ ]  b) NO...............................................................00  c) DON’T KNOW/DON’T REMEMBER 99 | |  |
| 308 | How many children do you have, who are alive now?  RECORD NUMBER | CHILDREN [ ][ ]  NONE 00 | | If no ⇒310 |
| 309 | How many of your living children are girls and how many are boys? | GIRLS ………………………………………...[ ]  BOYS …………………………………………[ ] | |  |
| *I would like to inform you again that all information you give will not be disclosed to anyone.* | | | | |
| 310 | I would like to ask you about your current pregnancy. At the time you became pregnant, did you want to become pregnant then, did you want to wait until later, did you want no (more) children, or did you not mind either way? | BECOME PREGNANT THEN 1  WAIT UNTIL LATER 2  NOT WANT CHILDREN 3  NOT MIND EITHER WAY 4  DON’T KNOW/DON’T REMEMBER 8  REFUSED/NO ANSWER 9 | |  |
| 311 | At the time you became pregnant, did your husband/partner want you to become pregnant then, did he want to wait until later, did he want no (more) children at all, or did he not mind either way? | BECOME PREGNANT THEN 1  WAIT UNTIL LATER 2  NOT WANT CHILDREN 3  NOT MIND EITHER WAY 4  DON’T KNOW/DON’T REMEMBER 8  REFUSED/NO ANSWER 9 | |  |
| 312 | Does your husband/partner stop you, encourage you, or have no interest in whether you receive antenatal care for your pregnancy? | STOP 1  ENCOURAGE 2  NO INTEREST 3  DON’T KNOW/DON’T REMEMBER 8  REFUSED/NO ANSWER 9 | |  |
| 313 | When you became pregnant, did your husband/partner have preference for a son, a daughter or did it not matter to him whether it was a boy or a girl? | SON 1  DAUGHTER 2  DID NOT MATTER 3  DON’T KNOW/DON’T REMEMBER 8  REFUSED/NO ANSWER 9 | |  |
| VERIFY THAT ADDITION ADDS UP TO THE SAME FIGURE. IF NOT, PROBE AGAIN AND CORRECT. | | | [] 303____ + [] 304_____ =.......... 302(a) | |

| **SECTION 4 HOUSEHOLD CHARACTERISTICS AND FINANCES** | | | | |
| --- | --- | --- | --- | --- |
| Now I would like to ask you some questions about the household that you live in. | | | | |
| 401 | What is the total number of persons living in your household?  (including domestic servants if they sleep 5 nights a week or more in the household and visitors if they have slept in the household for the past 4 weeks) | TOTAL NUMBER: ……… …….. | IF LIVES ALONE ⇒ 403 | |
| 402 | Who among these live in your household? | YES NO SOME OF THE TIME  FATHER AND/  OR MOTHER 1 2 3  FATHER IN-LAW AND/  OR MOTHER IN-LAW 1 2 3 |  | |
| 403 | Do you live in | YOUR OWN HOUSE (HOW MANY ROOMS________) 1  RENTED HOUSE (HOW MANY ROOMS_______) 2  RENTED ROOMS (HOW MANY ROOMS________) 3  OTHER (SPECIFY)..................................................________) 4 |  | |
| 404 | Who is head of your household? | I AM ……………………………………………….. 1  MY HUSBAND/PARTNER …...............................2  MY MOTHER-IN-LAW ….....................................3  MY FATHER-IN-LAW …......................................4  MY MOTHER …...................................................5  MY FATHER …....................................................6  MY BROTHER ….................................................7.  MY SISTER …....................... ..............8  OTHER RELATIVE ….........................................9  DON’T KNOW …...............................................10 |  | |
| 405 | Does your household have: | YES NO DK  a) TELEVISION 1 2 3  b) LANDLINE PHONE 1 2 3  c) MOBILE PHONE 1 2 3  d) REFRIGERATOR 1 2 3  e) COMPUTER 1 2 3  f) BANK ACCOUNT 1 2 3 |  | |
| 406 | Are you able to spend the money you earn how you want yourself, | YES ALL THE TIME 1  YES SOME OF THE TIME 2  YES RARELY 3  NO 4  REFUSE TO ANSWER 5 | |  |
| 407 | Do you have to give all or part of the money to your husband/partner? | NO 1  YES, GIVE PART TO HUSBAND/PARTNER 2  YES, GIVE ALL TO HUSBAND/PARTNER 3  DON’T KNOW 8  REFUSED/NO ANSWER 9 | |  |

| **SECTION 5 CURRENT OR MOST RECENT PARTNER** | | | | | | |
| --- | --- | --- | --- | --- | --- | --- |
| I would now like you to tell me a little about your husband/partner | | | | | | |
| 501 | What is the age of your husband/partner in years? | AGE IN YEAR [ ][ ]  DONT REMEMBER..................................... 99 | | |  | |
| 502 | What is the highest level of education your partner achieved? MARK HIGHEST LEVEL. | NEVER ATTENDED SCHOOL ………. …. 1  PRIMARY ……………………………………. 2  SECONDARY…………………………………. 3  ABOVE SECONDARY………………………… 4  NUMBER OF YEARS SCHOOLING [ ][ ]  DON’T KNOW/DON’T REMEMBER 98  REFUSED/NO ANSWER 99 | | |  | |
| 503 | What is your husband/partner’s occupation? | GOVERNMENT EMPLOYEE ............ .........1  PRIVATE COMPANY/ORGANISATION EMPLOYEE .................................................. 2  FARMER ......... ........... ......... ......... ............. 3  BUSINESS ......... ........... ......... ......... ......... 4  STUDENT ......... .......... ......... ......... ......... 5  UNEMPLOYED ......... .......... ......... ............. 6  OTHER (SPECIFY).......................................7 | | | |  |
| 504 | What is his religion? | NO RELIGION......... ......... ......... ......... 0  CATHOLIC ......... ......... ......... ...................1  LUTHERAN ......... ......... .............. .............2  PENTECOSTAL......... ......... . ......... .........3  SEVENTH DAY ADVENTIST ....... ......... 4  ANGLICAN ......... ......... ......... .............. 5  MUSLIM ......... ......... ......... ......................6  TRADITIONAL RELIGION ......... ...... ...... 7  OTHER (SPECIFY) …………………………….8  DON’T KNOW/DON’T REMEMBER............98  REFUSED/NO ANSWER ......... ......... .....99 | | | |  |
| 505 | How often does/did your husband/partner drink alcohol?  Every day or nearly every day  Once or twice a week  1–3 times a month  Occasionally, less than once a month  Never | EVERY DAY OR NEARLY EVERY DAY 1  ONCE OR TWICE A WEEK 2  1–3 TIMES IN A MONTH 3  LESS THAN ONCE A MONTH 4  NEVER 5  DON’T KNOW/DON’T REMEMBER 8  REFUSED/NO ANSWER 9 | | | | IF NEVER  ⇒508 |
| 506 | In the past 12 months (In the last 12 months of your last relationship), how often have you seen (did you see) your husband/partner drunk before having sex? Would you say every time, sometime or never? | EVERY TIME 1  SOMETIME 2  NEVER 3  DON’T KNOW/DON’T REMEMBER 8  REFUSED/NO ANSWER 9 | | | |  |
| 507 | In the past 12 months (In the last 12 months of your relationship), have you experienced any of the following problems, related to your husband/partner’s drinking?  Money problems  Family problems  Any other problems, specify. | a) MONEY PROBLEMS  b) FAMILY PROBLEMS  x) OTHER: _______________ | YES  1  1  1 | NO  2  2  2 | |  |
| 508 | Does/did your husband/partner ever use drugs?  Would you say:  Every day or nearly every day  Once or twice a week  1 – 3 times a month  Occasionally, less than once a month  Never | EVERY DAY OR NEARLY EVERY DAY 1  ONCE OR TWICE A WEEK 2  1 – 3 TIMES IN A MONTH 3  LESS THAN ONCE A MONTH 4  NEVER 5  IN THE PAST, NOT NOW 6  DON’T KNOW /DON’T REMEMBER 8  REFUSED/NO ANSWER 9 | | | |  |

| **SECTION 6 VIOLENCE SCREEN** | | | | | |
| --- | --- | --- | --- | --- | --- |
|  | When two people marry or live together, they usually share both good and bad moments. I would now like to ask you some questions about your husband/partner and how your husband/partner treats (treated) you.  If anyone interrupts us I will change the topic of conversation. I would again like to assure you that your answers will be kept secret, and that you do not have to answer any questions that you do not want to. May I continue? | | | | |
| 601 | In your relationship with your husband/partner, how often would you say that you quarrelled? Would you say rarely, sometimes or often? | RARELY ......... ......... ......... ......... ......... 1  SOMETIMES......... ......... ......... ......... 2  OFTEN ......... ......... ......... ......... ......... .........3  DON’T KNOW/DON’T REMEMBER ......... .....8  REFUSED/NO ANSWER ......... ......... ..........9 | | |  |
| 602 | The next questions are about things that happen to many women, and that your husband/partner may have done to you.  In the past 12 months, has your husband/partner done any of the following things to you: | YES  1 | NO  2 | DONT REMEMBER  3 | IF YES TO ANY  ⇒ 603  IF NO TO ALL  ⇒ SECTION 7 |
|  | Done things to scare or intimidate you on purpose (e.g. by the way he looked at you, by yelling or smashing things)? | 1 | 2 | 3 |  |
|  | Threatened to hurt you or someone you care about? | 1 | 2 | 3 |  |
|  | Hit you, slapped you, or thrown something at you that could hurt you? | 1 | 2 | 3 |  |
|  | Forced you or pressured you to have sexual intercourse when you did not want to? | 1 | 2 | 3 |  |
| 603 | If the respondent answers yes to any of the above, ask:  During this pregnancy, has your husband/partner done any of the following things to you: | YES  1 | NO  2 | DONT REMEMBER  3 |  |
|  | Done things to scare or intimidate you on purpose (e.g. by the way he looked at you, by yelling or smashing things)? | 1 | 2 | 3 |  |
|  | Threatened to hurt you or someone you care about? | 1 | 2 | 3 |  |
|  | Hit you, slapped you, or thrown something at you that could hurt you? | 1 | 2 | 3 |  |
|  | Forced you or pressured you to have sexual intercourse when you did not want to? | 1 | 2 | 3 |  |

| **SECTION 7 RESILIENCE** | | | | | | | | |
| --- | --- | --- | --- | --- | --- | --- | --- | --- |
| Now I would like to ask some questions about how you have felt over the last month. If a particular situation has not occurred recently, answer according to how you think you would have felt. | | | | | | | | |
|  | (GIVE SCALE AND SAY....use this scale of 1 to 5 to indicate how you felt or you would feel of the particular situation. It range from 1 which is true nearly all the time up to 5 which is not true at all. | True nearly all the time  1 | Often true  2 | Sometimes true  3 | Rarely true  4 | Not true at all  5 | Don’t know/No answer  9 |  |
| 701 | I am able to adapt when changes occur | 1 | 2 | 3 | 4 | 5 | 9 |
| 702 | I can deal with whatever comes my way | 1 | 2 | 3 | 4 | 5 | 9 |
| 703 | I try to see the humorous side of things when I am faced with problems. | 1 | 2 | 3 | 4 | 5 | 9 |
| 704 | Having to cope with stress can make me stronger. | 1 | 2 | 3 | 4 | 5 | 9 |
| 705 | I tend to bounce back after illness, injury, or other hardships. | 1 | 2 | 3 | 4 | 5 | 9 |
| 706 | I believe I can achieve my goals, even if there are obstacles. | 1 | 2 | 3 | 4 | 5 | 9 |
| 707 | Under pressure, I stay focused and think clearly. | 1 | 2 | 3 | 4 | 5 | 9 |
| 708 | I am not easily discouraged by failure. | 1 | 2 | 3 | 4 | 5 | 9 |
| 709 | I think of myself as a strong person when dealing with life’s challenges and difficulties. | 1 | 2 | 3 | 4 | 5 | 9 |
| 710 | I am able to handle unpleasant or painful feelings like sadness, fear, and anger. | 1 | 2 | 3 | 4 | 5 | 9 |  |

| **SECTION 8 COMPLETION OF INTERVIEW** | | | |
| --- | --- | --- | --- |
|  | We have now finished the interview. Do you have any comments, or is there anything else you would like to add? | |  |
|  | ______________________________________________________________________________________________________________________________________________________________________________________________________________________________________________________________________________________________________________________________________________________________________________________________________________________________________________________________________________________________________________________________________________________________________________________________________________________________________________________________________________________________________________________________________________________________________________________________________________________________________________________________________________________________________________________________________________________________________________________________________________________________________________________________________________________________________________________________________________________________________________________________________________________________________________________________________________________________________________________________________________________________________________________________________ | |  |
|  | Finally, do you agree that we may contact you again in 6 weeks’ time? | YES ......... ......... .........1  NO ......... ......... .........2 |  |
|  | I THINK YOU HAVE BEEN GIVEN A TENTATIVE DATE FOR THE NEXT INTERVIEW: REMIND ME OF THAT DATE:  DATE: __________________________  (Inform her that we may contact her two days before appointment )  ASK THE WOMAN WHERE SHE PREFERS THE NEXT INTERVIEW TO TAKE PLACE AND WRITE IT DOWN:  ASK HER IF IT IS OK THAT WE CALL AND/OR COME TO HER HOME IN CASE SHE FORGETS OUR INTERVIEW APPOINTMENT: | HOME.............................1  CLINIC............................2  YES ......... ......... ......... 1  NO ......... ......... ......... 2 |  |
|  |  |  |  |
|  |  |  |  |
|  | INFORM HER WHO HER CONTACT PERSON IS:  _______________________________________________ |  |  |

|  | | **FINISH ONE – IF RESPONDENT HAS DISCLOSED PROBLEMS/VIOLENCE** |  | |
| --- | --- | --- | --- | --- |
|  | | I would like to thank you very much for helping us. I appreciate the time that you have taken. I realize that these questions may have been difficult for you to answer, but it is only by hearing from women themselves that we can really understand about their health and experiences of violence.  From what you have told us, I can tell that you have had some very difficult times in your life. No one has the right to treat someone else in that way. However, from what you have told me I can see also that you are strong, and have survived through some difficult circumstances.  SOFT REFERRAL (LOW RISK CASES): Here is a list of organizations that provide support, legal advice and counselling services to women. Please do contact them if you would like to talk over your situation with anyone. Their services are free, and they will keep anything that you say private. You can go whenever you feel ready to, either soon or later on.  (IF SOFT REFFERAL INDICATE ACTIONS TAKEN BELOW )  HARD REFERRAL (HIGH RISK CASES): The violence you have told me about sounds quite serious. Is it OK with you if I refer you to our nurse counsellor who will help contact people who may be able to help you and ask them to contact you directly?  (IF HARD REFFERAL INDICATE ACTIONS TAKEN BELOW ) |  | |
|  | | **FINISH TWO - IF RESPONDENT HAS NOT DISCLOSED PROBLEMS/VIOLENCE** |  | |
|  | | I would like to thank you very much for helping us. I appreciate the time that you have taken. I realize that these questions may have been difficult for you to answer, but it is only by hearing from women themselves that we can really understand about women’s health and experiences in life. |  | |
| RECORD TIME OF END OF INTERVIEW: Hour [ ][ ] (24 h)  Minutes [ ][ ] | | | | |
| ASK THE RESPONDENT. How long did you think the interview lasted ?  Hours [ ] Minutes [ ][ ] | | | | |
| INTERVIEWER COMMENTS TO BE COMPLETED AFTER INTERVIEW | | | | |
|  | ________________________________________________________________________________________________________________________________________________________________________________________________________________________________________________________________________________________________________________________________________________________________________________________________________________________________________________________________________________________________________________________________________________________________________________________________________________________________________________________________________________________________________________________________________________________________________________________________________________________________________________________________________________________________________________________________________________________________________________________________________________________________________________________________________________________________________________________________________________________________________________________________________________________________________________________________________________________________________________________________________________________________________________________________________________________________________________________________________________________________________________________________________________________________________________________________________________________________________________________________________________________________________________________________________________________________________________________________ | | |  |
